# Impact of resilience and social support on long-term grief in cancer-bereaved siblings: an exploratory study

**DOI:** 10.1186/s12904-022-00978-5

**Published:** 2022-06-01

**Authors:** Omid Rasouli, Unni Karin Moksnes, Trude Reinfjell, Odin Hjemdal, Mary-Elizabeth Bradley Eilertsen

**Affiliations:** 1 Department of Public Health and Nursing, Faculty of Medicine and Health Sciences, Norwegian University of Science and Technology, Trondheim, Norway; 2 Department of Neuromedicine and Movement Science, Faculty of Medicine and Health Sciences, Norwegian University of Science and Technology, Trondheim, Norway; 3 Department of Psychology, Faculty of Social and Educational Sciences, Norwegian University of Science and Technology, Trondheim, Norway

**Keywords:** Bereaved families, Childhood cancer, Unresolved grief, Bereavement, Young adult

## Abstract

**Background:**

Bereavement research has mainly explored potential risk factors associated with adverse outcomes, and the role of protective factors has received less attention. More knowledge is needed about factors related to unresolved grief in bereaved siblings. This study aimed to assess grief adjustment and possible gender differences among bereaved young adults 2–10 years after losing a brother or sister to cancer. We also sought to explore how resilience and social support influenced their grief.

**Methods:**

A total of 99 young adults (18–26 years) who had lost a brother or sister to cancer between the years 2009 and 2014 were invited to participate in this Norwegian nationwide study. The study-specific questionnaire was completed by 36 participants (36.4%). Social support during the sibling's illness, after the death, and during the past year, in addition to grief and resilience, were measured.

**Results:**

Overall, the prevalence of unresolved grief was 47.2% among bereaved siblings, whereas 52.8% had worked through their grief. The level of having worked through grief and resilience was similar between male and female siblings. Bereaved siblings with higher Personal Competence reported lower unresolved grief.

**Conclusion:**

Approximately half of the young adults experience unresolved grief 2–10 years after losing a sibling to cancer. The findings also highlight the need for long-term support for bereaved siblings to help improve their resilience and better have worked through their grief.

## Introduction

Grief is the emotional and psychological reaction to the loss of significant someone, such as a parent or a child, and people often cope with their grief within 6–24 months after the loss [1]. However, others may develop extensive psychological distress and experience substantial consequences due to their loss [2]. Several theories for the grief process have suggested that bereaved individuals go through different stages or phases. Stroebe and Schut (1999) suggested the Dual Process Model of Coping with Bereavement to explain how bereaved individuals cope with their loss [3]. It is a dynamic model emphasizing continuous shifts between loss-oriented (e.g., crying and helplessness) and restoration-oriented experiences (e.g., family and financial demands) [4]. The dual process model includes the interaction between environmental factors and individual grief work [4].

A child’s death is one of the most traumatic events that siblings and parents may experience [5]. The bereavement process has been associated with developing psychological distress and emotional/behavioral problems, educational problems, and stress‐related medical conditions in bereaved siblings of children with cancer [6–9]. Physical health of surviving siblings is affected, particularly in the first six months after a sibling's death, regardless of the child’s gender [7]. The emotional problems experienced by cancer-bereaved siblings (e.g., loneliness, anxiety, and anger) seem to start during the dying child’s illness [6]. There are very few studies exploring the long-term adjustment of bereaved siblings [10]. One study observed unresolved grief in the majority (54%) of young adults 2–9 years after losing a sibling to cancer, and only 11% reported having worked through their grief completely [11]. The authors reported a lack of social support and more recent loss as contributing factors for not having worked through the grief [11]. Poor communication with family, friends, and healthcare staff increases the siblings’ risk of unresolved grief [12]. Lower social support was also associated with higher anxiety [8]. However, the role of social support and when it is most needed in this vulnerable group is unclear and needs to be investigated.

Although it is essential to reduce risk factors during stressful life events, focusing on protective factors and increasing positive outcomes is also crucial [13]. A protective factor can increase the likelihood of positive outcomes [14, 15]. However, bereavement and grief research has mainly explored potential risk factors associated with adverse effects, and the role of protective factors has received little attention [16]. Recent studies showed that a high level of resilience was a significant factor in a healthy adaptation to grief and lower psychological distress in parents who lost a child to cancer [17, 18]. Resilience is generally defined as a process reflecting positive adaptations despite experiencing substantial stressors/trauma such as childhood abuse or the loss of a significant person [19]. However, it is unknown which resilience factors are primarily associated with resolved grief in cancer-bereaved siblings. In general, positive adaptation is commonly defined as developing a good level of functioning in terms of health, social skills, and age-appropriate developmental tasks [20]. Indeed, evidence suggests that bereaved siblings also experience positive outcomes, including psychological gains, personal growth, and openness [6, 21–23]. Previous studies have reported mixed findings for demographic risk and protective factors. For example, some studies have found that female siblings reported poorer mental health and QoL [24] and more posttraumatic growth [25], whereas other studies have not observed similar trends [9, 26, 27]. Therefore, it is essential to identify both protective and risk factors contributing to unresolved grief to adequately meet their needs and promote positive outcomes in this vulnerable group.

To address the mentioned shortcomings, this study aimed to assess the level of having worked through grief and possible gender differences among bereaved young adults 2–10 years after losing a brother or sister to cancer. We also sought to explore how resilience and perceived social support influenced the grief of these siblings.

## Method

### Design

This population-based nationwide study had a retrospective, cross-sectional design. Information was obtained through a self-reported questionnaire from the participants. The study was approved by the Regional Committee for Medical Research Ethics Central Norway (2014/1997/REK Midt) and conducted according to the Declaration of Helsinki. All participants signed an informed consent form before participation.

### Participants and procedure

Children who died of cancer at ages < 24 years between January 2009 to December 2014 were identified through the Cancer Registry and confirmed by the Cause of Death Registry. A total of 113 siblings of those deceased children, who could be at any age at the time of the sibling’s death, were registered. Inclusion criteria were having lost a brother or sister to cancer from January 2009 to December 2014, having a valid postal address in Norway and speaking Norwegian. Fourteen persons had wrong postal addresses and were excluded. We sent an invitation letter describing the study’s objectives and a consent form to all bereaved siblings who met the inclusion criteria (*N* = 99). The questionnaire with a stamped return envelope was mailed to those bereaved siblings who agreed to participate. Finally, 36 siblings (11 males and 25 females) returned the completed questionnaire (36.4%).

### Assessment scales

A self-report study-specific questionnaire consisting of some standard scales and several questions specific for cancer-bereaved siblings was used in this study. This questionnaire was originally developed in Sweden specific for cancer-bereaved siblings and we used a Norwegian translation of that questionnaire [11, 28]. The questionnaire covers items regarding illness period, time after the sibling’s death, current life situation, and sociodemographic.

#### Grief

We measured the level of having worked through grief by a question: “To what extent do you think you have worked through your grief over your sibling’s death?” with four response options: “No, not at all,” “Yes, a little,” “Yes, a lot” and “Yes, completely.” This simple question has been used to measure grief in cancer-bereaved siblings and parents [11, 18, 29]. It was tested in face-to-face interviews to assure that the parents understood the item as intended, having worked through their grief or resolved their grief [29]. Also, it was validated against three questions adopted from the Inventory of Complicated Grief, i.e., “intense longing for the lost person,” “perceives life as empty without the lost person,” and “unable to trust others”; all three correlated strongly with our single-item question about grief resolution [11]. This item was dichotomized into Not at all/a little (had not worked through their grief) or enough/a lot (having worked through their grief) for regression analysis.

#### Resilience

Resilience Scale for Adolescents (READ) was used to measure resilience [30]. READ is a self-report scale with 28 items organized into five subscales: “Personal Competence,” “Social Competence,” “Structured Style,” “Family Cohesion,” and “Social Resources” [30]. All items are rated on a 5-point Likert scale ranging from “Strongly disagree” (1) to “Strongly agree” (5). The total READ score is the sum scores of the subscales; higher scores indicate higher levels of protective qualities associated with resilience within each of the subscales. High reliability and validity have been reported for the five subscales assessed by the READ [31]. READ was rated as the best scale for use on adolescents in a methodological review [32]. “Personal Competence” measures an individual’s levels of self-esteem, self-acceptance, self-efficacy, determination, hope, realistic life orientation, and ability to follow daily routines as planned. “Social Competence” refers to extraversion, social skills, humor, ability to start conversations, and flexibility in social environments. “Structured Style” measures the level of preference in which individuals plan and structure their daily routines. “Family Cohesion” evaluates the level of shared values in the family, the family’s ability to maintain a positive perspective, and there is also the perception of social support. “Social Resources” assesses the perception of access and external support availability, such as friends [30].

#### Social support

We measured perceived social support in general at three different time points: (1) during sibling’s illness period, (2) after the sibling’s death, and (3) during the past year. The first question: “To what extent did your need for social support get satisfied during your sibling’s illness period?” with three response alternatives “Not at all,” “Partially,” and “A lot.” This item was dichotomized into Not at all/partially (0) or a lot (1) for regression analysis.

The next question was “To what extent did your need for social support get satisfied after your sibling’s death?’ with four response alternatives “Not at all,” “A little,” “Enough,” and “A lot.” This item was dichotomized into Not at all/a little (0) or enough/a lot (1) for regression analysis. The last question was “To what extent did your need for social support get satisfied during the last year?’ with four response alternatives “Not at all,” “A little,” “Enough,” and “A lot.” For regression analysis, this item was dichotomized into Not at all/a little (0) or enough/a lot (1).

### Statistical analysis

The statistical analyses were performed by SPSS software (Version 27). Descriptive statistics were used to characterize the included participants. Normal distributions of data were tested using the Shapiro–Wilk test and residual plot assessment. Continuous variables were presented as mean and standard deviation (SD), and categorical variables as frequencies and percentages.

Demographic variables were compared between the genders using Chi-square or Fisher’s exact tests for categorical variables and independent t-tests for continuous variables. Fisher’s exact test was performed when the expected values were too low. Binary ordinal logistic regression was used to assess how well independent variables explain the dependent variable. In the first step, the associations between having worked through grief and each independent variable (social support items, resilience, and demographic variables) were explored separately using univariate logistic regression. Then, significant factors were put into a model and analyzed using multivariate logistic regression. The alpha level of significance was set at *p* < 0.05.

## Results

Participants were predominantly females currently studying, from big cities, and living alone. Participants’ age at the time of loss was 9–20.5 years, and at the time of the survey, 18–26 years. The mean age of their deceased siblings at the time of diagnosis and death was 13.4 (SD = 6.6) and 17.2 (SD = 4.9) years, respectively. The range of time since the loss was 2.5–10 years. Table 1 displays the sociodemographic information.

**Table 1:** Characteristics of the cancer-bereaved siblings (*n* = 36)

	*n* (%)
**Age at inclusion (years)** ^**a**^	22.6 (2.3)
**Age at loss (years)** ^**a**^	15.9 (2.5)
**Time since loss (years)** ^**a**^	6.9 (2.4)
**Illness duration (years)** ^**a**^	3.8 (4.5)
**Death in close family in the last year (Yes)**	10 (27.8%)
**Sex**	
Female	25 (69.4%)
Male	11 (30.6%)
**Deceased sibling’s gender**	
Female	20 (55.6%)
Male	16 (44.4%)
**Place of live**	
Rural area	5 (13.9%)
Small town	8 (22.2%)
Big city	23 (63.9%)
**Living with**	
Living independently	22 (61.1%)
Living with partner	5 (13.9%)
Living with parents	9 (25%)
**Education**	
Primary and lower secondary school	6 (16.7%)
High school	19 (52.8%)
Technical college	3 (8.3%)
College/university (3 years)	8 (22.2%)
**Work situation**	
Employed	11 (30.6%)
Studies	23 (63.9%)
Unemployed	2 (5.5%)

*Note*. ^a^ mean (standard deviation)

### Gender comparisons

**Fig. 1:**
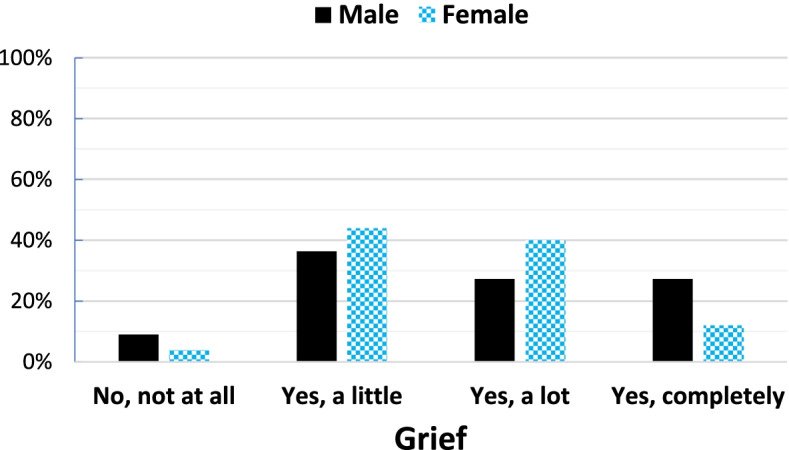
The extent of having worked through the grief 2–10 years after losing a brother/sister to cancer for each sex

Male participants (14.4 ± 2.7) were significantly younger at their sibling’s death than the females (16.5 ± 2.1; t (34) = -2.5, *p* = 0.02). There was a longer time since the loss for males (8.6 ± 2.1) compared to the females (6.2 ± 2.2; t (34) = 3.03, *p* = 0.005). Overall, 47.2% of the bereaved siblings had worked through their grief either “not at all” or “a little” at the investigation time, whereas 52.8% had worked through their grief “a lot” or “completely.” Fig. 1 illustrates the levels of having worked through grief for male and female siblings. A Chi-square test for independence indicated no significant association between gender and grief, χ2 (1, *n* = 36) = 0.02, *p* = 0.89, *phi* = -0.02. Table 2 lists the resilience subscales in each gender. There were no significant differences between the genders regarding the resilience subscales. Table 3 demonstrates perceived social support in participants. As shown in Table 3, most participants perceived enough/a lot of social support during the past year. However, the majority perceived not at all/little support during their sibling’s illness. Chi-square tests indicated no significant differences between genders regarding perceived social support (Table 3).

**Table 2:** Resilience scores for each subscale in the participants (*n* = 36)

Scale (Number of items)	Male (*n *= 11)	Female (*n* = 25)	*t* (34)	*p*-value*
**Personal Competence (8)**	3.5 (0.8)	3.5 (1.0)	0.17	0.856
**Social Competence (5)**	4.0 (0.7)	3.9 (0.7)	-0.25	0.791
**Structured Style (4)**	3.1 (0.6)	3.2 (0.7)	0.25	0.806
**Family Cohesion (6)**	4.1 (0.7)	4.1 (0.7)	-0.21	0.835
**Social Resources (5)**	4.3 (0.9)	4.6 (0.5)	1.10	0.277
**READ Total (28)**	3.8 (0.6)	3.9 (0.5)	0.23	0.817

Values are shown as mean (standard deviation)

^*^ *p*-values are from an independent-samples t-test to compare resilience scores between males and females. Degrees of freedom (df) was 34 for all independent-samples t-tests

**Table 3:** Perceived social support in the participants and tests of sex differences in perceived social support

Timepoint	Male (*n* = 11)		Female (*n* = 25)		Chi-square test	
	not at all/little	enough/a lot	not at all/little	enough/a lot	**χ2 (1, ** ***n*** ** = 36)**	***p*** **-value**
***During sibling’s illness***	7 (63.6)	4 (36.4)	18 (72)	7 (28)	0.25	0.62
***After sibling’s death***	5 (45.5)	6 (54.5)	9 (36)	16 (64)	0.29	0.59
***During the past year***	2 (18.2)	9 (81.8)	6 (24)	19 (76)	0.15	0.70

*Note*. Values are shown as numbers (%)

### Regression analysis

Table 4 presents unadjusted and adjusted ORs for the variables predicting having worked through grief in bereaved siblings. First, every item was analyzed separately as an independent variable and grief as the dependent variable and reported as unadjusted ORs. The univariate logistic regression analyses showed that only two variables, social support after a sibling’s death and personal competence, were significantly associated with having worked through grief (*p* < 0.05). Then, only these two variables were put into a model as independent variables and grief as the dependent variable and analyzed by multivariate logistic regression analysis while controlling for gender and time since the loss. This model significantly predicted having worked through grief in the participants (Pseudo R^2^: 44%, *p* < 0.007).

**Table 4:** Summary of binary logistic regression analysis of having worked through grief as an outcome variable in the bereaved siblings

	**Unadjusted OR (95% CI) ** ^**a**^	***p-*** **value ** ^**b**^	**Adjusted OR (95% CI)**	***p-*** **value ** ^**b**^
**Sex (females)**	1.11 (0.27, 4.59)	0.888	1.05 (0.12, 9.12)	0.963
**Age at inclusion (years)**	1.06 (0.80, 1.41)	0.678		
**Age at sibling’s death (years)**	1.14 (0.81, 1.40)	0.670		
**Time since loss (years)**	1.03 (0.76, 1.37)	0.869	1.12 (0.73, 1.74)	0.603
**Sibling’s illness duration**	0.87 (0.71, 1.05)	0.149		
**Support during illness (code = 0)**	3.39 (0.73, 15.90)	0.121		
**Support after death (code = 0)**	9.78 (2.01, 47.68)	0.005	6.34 (1.09, 36.70)	0.039
**Support in last year (code = 0)**	4.64 (0.79, 27.25)	0.090		
**Personal Competence**	3.47 (1.33, 9.09)	0.011	3.01 (1.05, 9.09)	0.041
**Social Competence**	2.45 (0.82, 6.15)	0.115		
**Structured Style**	1.37 (0.52, 3.61)	0.523		
**Family Cohesion**	2.76 (0.86, 8.84)	0.088		
**Social Resources**	4.33 (0.86, 21.81)	0.076		
			χ^2^_(4)_ 14.01, *p* < 0.007, Pseudo R^2^: 44%	

*OR* Odds ratio, *95% CI* 95% confidence interval

^a^Every single item was analyzed separately as an independent variable and grief as the dependent variable

[Code = 0]: Reference outcome

^b^Each reported *p*-value is associated with a chi-square test with df = 1

## Discussion

There is limited research on bereaved young adult siblings compared to research on bereaved parents or spouses. In the present study, we primarily investigated grief adjustment and possible gender differences in bereaved siblings 2–10 years after losing a sibling to cancer and how resilience and social support influenced the grief in the cancer-bereaved siblings. The findings of this study showed that approximately half (47.2%) of bereaved young adults had not worked through their grief even 2–10 years after the loss of a brother/sister to cancer. However, the prevalence of unresolved grief was similar between the female and male bereaved siblings. This finding confirms the study by Sveen et al. (2014), who found unresolved grief over the sibling’s death in most (54%) of cancer-bereaved siblings even after 2–9 years [11]. Although most individuals resolve their grief within two years after the loss [1], grief in adolescents and children is considered to be different from that in adults [33]. Individuals usually have the longest relationship with their siblings, even longer than relationships with their children or parents. The siblings’ bond is also considered one of the most significant in people’s lives [34]. The effects of a sibling’s death during childhood remain throughout the bereaved sibling’s lifetime [35]. In one study, almost all bereaved young adults reported that the loss still affected them even 12 years after the loss of a sibling to cancer [36]. Therefore, having worked through grief may be challenging, particularly during the vulnerable developmental stage of being a child or teenager. In this study, participants were aged 9–20.5 years when they lost their siblings. It is noteworthy that 5.6% of siblings reported that they had not worked through their grief at all, indicating a possible complicated grieving process. These individuals may need extra help and social support.

Bereaved siblings of children with cancer are identified as at higher risk for developing psychological, emotional/behavioral, and educational problems [6–9]. Nevertheless, bereaved siblings also experience positive outcomes post-loss, such as personal growth and openness [21–23, 36]. It is essential to employ protective factors during stressful life events to increase positive outcomes [14]. Resilience has been suggested as a central protective factor for mental health and having worked through grief in cancer-bereaved parents [17, 18]. To our knowledge, this is the first study investigating the level of resilience and its impact on grief among cancer-bereaved siblings. The results of the READ scale indicate similar resilience scores (range: 3.2—4.5) among the participants in the present study compared to unbereaved population scores [30].

Moreover, there were no significant differences in the resilience subscales between the genders. Only “Personal competence” was significantly associated with grief, indicating that individuals with higher personal competence scores had coped better with their grief (OR: 3.01). Personal competence is attributed to levels of self-esteem, self-acceptance, self-efficacy, determination, hope, realistic life orientation, and the ability to follow daily routines as planned [30]. The dual-process model also suggests that grief is worked through by finding a balance between dealing with grief in parallel with moving forward in life [4]. Therefore, it is beneficial for bereaved siblings to follow their daily routines and plan for their future. However, some individuals may need professional help to move forward in their life. A family bereavement program has been suggested as a successful intervention to help promote resilience in bereaved families [37].

The literature underlines that social support facilitates the grieving process [38] and lack of social support is a risk factor for adverse bereavement outcomes [39]. For example, lower perceived social support is associated with more psychological problems and unresolved grief in adolescent and young adult bereaved siblings [8, 11, 40]. We retrospectively measured social support measured at three different time points: (1) during sibling’s illness period, (2) after sibling’s death, and (3) during the past year. Among these three stages, only perceived social support after a sibling’s death was a significant factor for grief in the bereaved siblings, indicating that those satisfied with received support (i.e., enough or much support) had considerably coped better with their grief. This finding highlights the importance of social support after losing a sibling to cancer. This vulnerable group needs to receive both social family-based and hospital-based social support after death, as previously recommended [8]; however, it seems this group is overlooked by parents and healthcare professionals [41]. Bereaved siblings reported being alone with their feelings and dissatisfaction in the extended family [7, 8]. Thompson et al. (2011) reported family is an essential resource for social support in bereaved parents and siblings from 6 to 19 months after losing a child to cancer [42].

Pediatric oncology units play an important role in supporting families during palliation and bereavement periods. Accordingly, a bereavement program should include formal support services, supportive contact from hospital staff during the palliation phase and following the child’s death, and the opportunity to connect with other cancer-bereaved families to receive helpful information and support [43]. Moreover, school-based social support from friends, peers, and teachers has been reported to facilitate bereaved siblings’ adjustment [7, 44]. Similarly, Nolbris and Hellström (2005) reported friends as a valuable source of social support to help bereaved siblings cope with their grief [7].

Therefore, bereaved siblings should be offered psychosocial support such as cognitive behavioral therapy and counseling sessions to strengthen siblings in their transition to a new reality and support them with helpful coping strategies [45]. Clinical care should identify at‐risk siblings for developing unresolved and complicated grief and provide specific interventions to support those bereaved individuals in the short and long term [46].

### Strength and limitation

The main strength of the present study was including nationwide data. Nevertheless, we acknowledge some limitations of this study. We had a small sample size (n:36) with a low response rate (36.4%), which may have resulted in missing individuals with greater problems. Therefore, the results of this study should be interpreted with caution. However, Norway is a small country with just 5.4 million inhabitants. Also, this study was performed in the cultural setting of Norway with a relatively homogeneous population; the results may not be generalizable to other populations. Furthermore, grief and social support were only measured with one item. We cannot claim causality due to this study’s cross-sectional design, and resilience may be the product and not the cause of grief resolution. The retrospective process may have affected the participants’ responses. Moreover, factors related to grief, such as pre-existing mental illness and time spent with the patient during the final weeks, were not included in the analysis. Thus, future studies are warranted to provide more information regarding the casualty and develop effective evidence‐based knowledge for this population.

## Conclusion

The findings of this study show that over half of bereaved young adults had worked through their grief 2–10 years after the loss of a brother/sister to cancer. However, 47.2% of them had not worked through their grief, and this is a group that needs professional help and interventions. No difference was found in the level of having worked through grief between the female and male bereaved siblings. Those who reported higher levels of resilience (i.e., personal competence) and/or were satisfied with social support after their siblings’ death reported significantly having worked through their grief. However, the findings highlight that this vulnerable group may need long‐term support to help strengthen their resilience and meet their needs to adjust to life after a loss properly.

## Data Availability

The datasets generated and/or analyzed during the current study are not publicly available due to some personal information but are available from the corresponding author on reasonable request.

## Abbreviations

OR: Odds ratio
READ: Resilience Scale for Adolescents
SD: Standard deviation

## References

[1] Maciejewski PK, Zhang B, Block SD, Prigerson HG (2007). An empirical examination of the stage theory of grief. JAMA.

[2] Kristensen P, Dyregrov K, Dyregrov A (2017). What distinguishes prolonged grief disorder from depression?. Tidsskrift for den Norske laegeforening: tidsskrift for praktisk medicin, ny raekke.

[3] Stroebe M, Schut H (1999). The dual process model of coping with bereavement: rationale and description. Death Stud.

[4] Stroebe M, Schut H (2010). The dual process model of coping with bereavement: a decade on. Omega (Westport).

[5] Endo K, Yonemoto N, Yamada M (2015). Interventions for bereaved parents following a child's death: a systematic review. Palliat Med.

[6] Alderfer MA, Long KA, Lown EA, Marsland AL, Ostrowski NL, Hock JM, Ewing LJ (2010). Psychosocial adjustment of siblings of children with cancer: a systematic review. Psychooncology.

[7] Nolbris M, Hellstrom AL (2005). Siblings' needs and issues when a brother or sister dies of cancer. J Pediatr Oncol Nurs.

[8] Eilertsen ME, Eilegard A, Steineck G, Nyberg T, Kreicbergs U (2013). Impact of social support on bereaved siblings' anxiety: a nationwide follow-up. J Pediatr Oncol Nurs.

[9] McDonald FEJ, Patterson P, Tindle R (2020). What young people need when a family member dies of cancer. Support Care Cancer.

[10] van der Geest IM, Darlington AS, van den Heuvel-Eibrink MM (2015). Re: Long-term psychosocial outcomes among bereaved siblings of children with cancer. J Pain Symptom Manage.

[11] Sveen J, Eilegard A, Steineck G, Kreicbergs U (2014). They still grieve-a nationwide follow-up of young adults 2–9 years after losing a sibling to cancer. Psychooncology.

[12] Lovgren M, Sveen J, Nyberg T, Eilegard Wallin A, Prigerson HG, Steineck G, Kreicbergs U (2018). Care at end of life influences grief: a nationwide long-term follow-up among young adults who lost a brother or sister to childhood cancer. J Palliat Med.

[13] Friborg O, Hjemdal O, Rosenvinge JH, Martinussen M (2003). A new rating scale for adult resilience: what are the central protective resources behind healthy adjustment?. Int J Methods Psychiatr Res.

[14] Stroebe MS, Folkman S, Hansson RO, Schut H (2006). The prediction of bereavement outcome: development of an integrative risk factor framework. Soc Sci Med.

[15] Rasouli O, Vegsund HK, Eilegard Wallin A, Hjemdal O, Reinfjell T, Moksnes UK, et al. Bereaved parents' quality of life: resilience and professional support. BMJ Support Palliat Care. 2021;bmjspcare-2020-002840. 10.1136/bmjspcare-2020-002840. PMID: 34732472. 10.1136/bmjspcare-2020-002840 34732472

[16] Jaaniste T, Coombs S, Donnelly TJ, Kelk N, Beston D (2017). Risk and resilience factors related to parental bereavement following the death of a child with a life-limiting condition. Children (Basel).

[17] Vegsund HK, Reinfjell T, Moksnes UK, Wallin AE, Hjemdal O, Eilertsen MB (2019). Resilience as a predictive factor towards a healthy adjustment to grief after the loss of a child to cancer. PLoS One.

[18] Rasouli O, Aarseth Bo M, Reinfjell T, Moksnes UK, Eilertsen MB (2021). Protective and risk factors associated with psychological distress in cancer-bereaved parents: a cross-sectional study. Eur J Oncol Nurs.

[19] Masten AS (2001). Ordinary magic. Resilience processes in development. Am Psychol.

[20] Luthar SS (2006). Resilience in development: a synthesis of research across five decades. Developmental psychopathology: Risk disorder, and adaptation. edn. Edited by Cicchetti D, Cohen DJ.

[21] Martinson IM, Campos RG (1991). Adolescent bereavement: Long-term responses to a sibling's death from cancer. J Adolesc Res.

[22] Akard TF, Skeens MA, Fortney CA, Dietrich MS, Gilmer MJ, Vannatta K, Barrera M, Davies B, Wray S, Gerhardt CA (2019). Changes in siblings over time after the death of a brother or sister from cancer. Cancer Nurs.

[23] Foster TL, Gilmer MJ, Vannatta K, Barrera M, Davies B, Dietrich MS, Fairclough DL, Gerhardt CA (2012). Changes in siblings after the death of a child from cancer. Cancer Nurs.

[24] Havermans T, Croock ID, Vercruysse T, Goethals E, Diest IV (2015). Belgian siblings of children with a chronic illness: Is their quality of life different from their peers?. J Child Health Care.

[25] Kamibeppu K, Sato I, Honda M, Ozono S, Sakamoto N, Iwai T, Okamura J, Asami K, Maeda N, Inada H (2010). Mental health among young adult survivors of childhood cancer and their siblings including posttraumatic growth. J Cancer Surviv.

[26] McDonald FE, Patterson P, White KJ, Butow P, Bell ML (2015). Predictors of unmet needs and psychological distress in adolescent and young adult siblings of people diagnosed with cancer. Psychooncology.

[27] Canter KS, Wu YP, Odar Stough C, Parikshak S, Roberts MC, Amylon MD (2014). The relationship between attitudes toward illness and quality of life for children with cancer and healthy siblings. J Child Fam Stud.

[28] Vegsund H-K, Rannestad T, Reinfjell T, Moksnes U, Wallin A, Eilertsen M-E (2018). Translation and linguistic validation of a swedish study-specific questionnaire for use among Norwegian parents who lost a child to cancer. Soc Sci.

[29] Lannen PK, Wolfe J, Prigerson HG, Onelov E, Kreicbergs UC (2008). Unresolved grief in a national sample of bereaved parents: impaired mental and physical health 4 to 9 years later. J Clin Oncol.

[30] Hjemdal O, Friborg O, Stiles TC, Martinussen M, Rosenvinge JH (2017). A new scale for adolescent resilience: grasping the central protective resources behind healthy development. Meas Eval Couns Dev.

[31] Moksnes UK, Haugan G (2018). Validation of the Resilience Scale for Adolescents in Norwegian adolescents 13–18 years. Scand J Caring Sci.

[32] Windle G, Bennett KM, Noyes J (2011). A methodological review of resilience measurement scales. Health Qual Life Outcomes.

[33] Sood AB, Razdan A, Weller EB, Weller RA (2006). Children's reactions to parental and sibling death. Curr Psychiatry Rep.

[34] Cicirelli V (2013). Sibling relationships across the life span: Springer Science & Business Media.

[35] Machajewski V, Kronk R (2013). Childhood grief related to the death of a sibling. Jnp-J Nurse Pract.

[36] Rosenberg AR, Postier A, Osenga K, Kreicbergs U, Neville B, Dussel V, Wolfe J (2015). Long-term psychosocial outcomes among bereaved siblings of children with cancer. J Pain Symptom Manage.

[37] Sandler IN, Wolchik SA, Ayers TS, Tein JY, Luecken L (2013). Family Bereavement Program (FBP) approach to promoting resilience following the death of a parent. Fam Sci.

[38] Kreicbergs UC, Lannen P, Onelov E, Wolfe J (2007). Parental grief after losing a child to cancer: impact of professional and social support on long-term outcomes. J Clin Oncol.

[39] Stroebe M, Stroebe W, Schut H (2001). Gender differences in adjustment to bereavement: an empirical and theoretical review. Rev Gen Psychol.

[40] Pedro IC, Galvao CM, Rocha SM, Nascimento LC (2008). Social support and families of children with cancer: an integrative review. Rev Lat Am Enfermagem.

[41] Steele AC, Kaal J, Thompson AL, Barrera M, Compas BE, Davies B, Fairclough DL, Foster TL, Jo Gilmer M, Hogan N (2013). Bereaved parents and siblings offer advice to health care providers and researchers. J Pediatr Hematol Oncol.

[42] Thompson AL, Miller KS, Barrera M, Davies B, Foster TL, Gilmer MJ, Hogan N, Vannatta K, Gerhardt CA (2011). A qualitative study of advice from bereaved parents and siblings. J Soc Work End Life Palliat Care.

[43] deCinque N, Monterosso L, Dadd G, Sidhu R, Macpherson R, Aoun S (2006). Bereavement support for families following the death of a child from cancer: experience of bereaved parents. J Psychosoc Oncol.

[44] Howard Sharp KM, Russell C, Keim M, Barrera M, Gilmer MJ, Foster Akard T, Compas BE, Fairclough DL, Davies B, Hogan N (2018). Grief and growth in bereaved siblings: interactions between different sources of social support. Sch Psychol Q.

[45] Gerhardt CA, Lehmann V, Long KA, Alderfer MA (2015). Supporting siblings as a standard of care in pediatric oncology. Pediatr Blood Cancer.

[46] Warnick AL (2015). Supporting youth grieving the dying or death of a sibling or parent: considerations for parents, professionals, and communities. Curr Opin Support Palliat Care.

